# Community Perceptions of Zinc Biofortified Flour during an Intervention Study in Pakistan

**DOI:** 10.3390/nu14040817

**Published:** 2022-02-15

**Authors:** Usman Mahboob, Marena Ceballos-Rasgado, Victoria Hall Moran, Edward J. M. Joy, Heather Ohly, Mukhtiar Zaman, Nicola M. Lowe

**Affiliations:** 1 Institute of Health Professions Education and Research, Khyber Medical University, Peshawar 25100, Pakistan; usman.mahboob@kmu.edu.pk; 2 Centre for Global Development, University of Central Lancashire, Preston PR1 2HE, UK; mceballosrasgado@uclan.ac.uk (M.C.-R.); vlmoran@uclan.ac.uk (V.H.M.); hohly1@uclan.ac.uk (H.O.); 3 Department of Population Health, London School of Hygiene & Tropical Medicine, London WC1E 7HT, UK; edward.joy@lshtm.ac.uk; 4 Rothamsted Research, Department of Sustainable Agricultural Sciences, Harpenden, Hertfordshire AL5 2JQ, UK; 5 Department of Pulmonology, Rehman Medical Institute, Peshawar 25000, Pakistan; mza38@hotmail.com

**Keywords:** zinc, biofortification, deficiency, Pakistan, qualitative, community

## Abstract

Zinc-biofortified flour may be a cost-effective approach to improve zinc status of populations in low-resource settings. The success of biofortification programmes is subject to acceptability and uptake by consumers. This study explored community leaders’ and community members’ (*n* = 72) experiences and attitudes towards the flour provided during a cluster randomised controlled trial of zinc biofortified wheat in rural Pakistan (BiZiFED2). Focus group discussions (*n* = 12) were conducted and thematic analysis applied using an inductive, semantic, contextualist approach. Five themes were identified: (1) Contribution to food security; (2) Better sensory and baking properties than local flour; (3) Perceived health benefits; (4) Willingness to pay for the flour; and (5) Importance of trusted promoters/suppliers. Although the participants were blind to whether they had received control or biofortified flour, referred to collectively as “study flour”, the results indicated that the study flour performed well in terms of its taste and bread making qualities, with no adverse reports from participants in either arm of the BIZIFED2 RCT. Participants suggested that they would buy the biofortified wheat if this was available at a fair price due to perceived health benefits, reporting positive sensory characteristics and cooking attributes when compared to the flour available in the local markets. Overall, there was a positive reception of the programme and flour among the participants, and members of the community hoped for its continuation and expansion.

## 1. Introduction

Zinc deficiency is estimated to affect approximately 17% of the world’s population [1,2], with much higher prevalence in low- and middle-income countries [3]. Zinc deficiency is a contributing factor in the pathophysiology of diarrhoea-, pneumonia- and malaria-related morbidity and mortality and impaired growth in young children. Supplementation of children with zinc has been found to reduce diarrhoea incidence and morbidity and marginally improve height [4]. Children and women of reproductive age are groups at greater risk of suffering micronutrient deficiencies due to increased nutrient requirements [5]. In Pakistan, the recent National Nutrition Survey reported that the prevalence of zinc deficiency in children under 5 years of age and women of reproductive age was 18.6% and 22.1%, respectively [6]. Populations living in rural socio-economically deprived communities may be at higher risk of micronutrient deficiencies as they typically have low diet diversity and a high prevalence household food insecurity [7]. We have found that approximately 30% of women of reproductive age living in the brick kiln areas around Peshawar had plasma zinc concentrations that fall below the cut-off value of 660 µg/L for adequacy [8]. Effective actions to eliminate hidden hunger among the most vulnerable groups are required to achieve the Sustainable Development Goal 2 of Zero hunger by 2030 [9]. Current strategies to improve micronutrient intake include supplementation, fortification, diet diversification and biofortification. All four strategies could lead to positive health outcomes; however, their effectiveness will depend on the context in which these strategies are being set, compliance, quality control and the resources available [10].

Biofortification is the process by which the nutritional quality of food crops is improved through genetic engineering, conventional breeding or pre-harvest agronomic practices [11]. These processes can enhance the nutrient concentrations of staple crops which are consumed in large quantities among many food-insecure populations that have little or no access to fruits and vegetables, animal-source foods, micronutrient supplements or fortified foods [12]. For a biofortification strategy to be successful, the micronutrient density of the targeted food needs to have a measurable and significant impact on nutritional status of the populations consuming it and food crops need to have good acceptability and adoption among both producers and consumers [13,14]. From a producers perspective, considerations of acceptability and adoption may relate to the disease resistance, drought tolerance, marketability and costs of production of the crop, whilst consumers may be influenced by sensory characteristics (i.e., colour, flavour, texture, solubility and viscosity), affordability, among many other factors [13].

A “high-Zn” wheat variety (Zincol-2016) was developed by Pakistan’s National Agriculture Research System (NARS) using traditional breeding techniques with support from HarvestPlus and was released by the Pakistan Agriculture Research Council (PARC) in 2016. This variety of wheat, when grown with zinc fertilisers, contains more zinc per kilogram of grain compared with traditional varieties [15]. The Biofortified Zinc Flour to Eliminate Deficiency (BiZiFED) [16] and its continuation BiZiFED2 project [8], is a trial which aims to examine the impact of consuming zinc-biofortified flour (from Zincol-2016 grown with zinc fertilisers) as a strategy to reduce zinc and iron deficiencies amongst adolescent girls and children. Since the success of the biofortification programmes and their potentiality to be scaled up are dependent to the acceptability of the biofortified crops, it is important to understand the socio-cultural factors and market systems that affect the sustainable uptake of biofortified wheat in Pakistan. The aim of this qualitative study was to explore the experiences of the study flour provided during a cluster RCT of zinc biofortified wheat in rural Pakistan from the perspective of the consumers (i.e., community members and community leaders and also their attitudes towards the concept of biofortification). 

## 2. Materials and Methods

### 2.1. Study Setting and Design

This study is part of the BiZiFED2 project (Biofortification with Zinc and Iron for Eliminating Deficiency), funded by the United Kingdom Research and Innovation (UKRI) Global Challenge Research Fund (GCRF), which started in April 2019 and will conclude in March 2022. BiZiFED2 included a cluster-randomised, double-blind, controlled trial designed to test the effectiveness of zinc biofortified wheat flour for improving zinc status of adolescent girls and children in rural Pakistan [8]. The study was conducted in two neighbouring remote communities located 30 km east of Peshawar city in the Khyber Pakhtunkhwa Province of Pakistan. These are impoverished rural communities, with livelihoods dependent on employment in nearby brick kilns and small-scale agriculture, and household incomes are typically less than USD 10 per day [17]. Wheat is the staple in this area and diets are low in animal-source foods. The wheat flour is purchased locally from the local market according to household requirement and consumed as paratha, chapatti and roti. This area was formerly part of a tribal society with traditional and conservative values. Decisions are made on behalf of the community by Jirga—groups of male elders from each village who are trusted and respected by the community and whose decisions filter down to the household level. Likewise, problems or concerns at the household level are escalated, discussed and resolved by the Jirga. Involving the Jirga at regular intervals during the development and conduct of our work helped to ensure that our methods were feasible and culturally acceptable [18]. Our research team included a local NGO (Abaseen Foundation Pakistan) that has a good working relationship with these communities and is engaged in ongoing education and health projects [15,17,19]. The protocols for the study were co-developed by the authors following the guidelines set out by the Global Code of Conduct for Research in Resource-Poor Settings [20] and sensitive to the local context and norms of the Pashtun culture. In accordance with local context, male and female research assistants were employed to collect data from gender-specific groups.

### 2.2. Sampling and Selection of Study Participants

Twelve focus group discussions (FGDs) were conducted: two FGDs with male community members, two FGDs with female community members, and two FGDs with male Jirga members in each community. FGDs were planned with 6 participants in each group to enable a sufficiently rich discussion whilst adhering to Pakistan Government COVID-19 restrictions at the time of data collection. A purposive sampling technique was used to select the FGD participants. Potential participants were eligible if: they were members of households participating in the BiZiFED2 RCT or were part of the community Jirga; were over the age of 18; and could willingly give informed consent. The community members were selected from the 500 households that participated in the BiZiFED2 RCT and further detail of eligibility to the BiZiFED2 RCT has been described elsewhere [8]. The selection of participants for the FGDs was conducted by the BiZiFED2 RCT management team who identified individuals who were willing to speak openly as engaged members of the community. Given that participants and researchers on the BiZiFED2 RCT were blind to the allocation arm, the participants invited to the FGDs could have been members of households allocated to either the BiZiFED2 RCT intervention arm (*n* = 250, provided with flour milled from the biofortified strain of wheat (Zincol-2016)), or the control arm BiZiFED2 RCT (*n* = 250, given flour milled from a standard variety of wheat (Galaxy-2013). An earlier randomised controlled efficacy study conducted by the same research group reported that Zincol-2016 wheat, grown under optimal conditions of zinc fertiliser application specifically for this study, was able to achieve a mean zinc concentration of 49.3 mg/kg compared to 22.2 mg/kg in Galaxy-2013 grain [21]. Further information about the study flour (biofortified and control) and its production and distribution has been described in the BiZiFED2 study protocol [8].

### 2.3. Ethical Considerations

Literate participants were given a Participant Information Sheet, a consent form and COVID-19 Research Participant Pre-Visit Check form (all in Urdu) to identify individuals who may be at greater risk of transmitting the virus during the recruitment process. Participants were asked not to attend the FGD if they had recently been exposed to someone with COVID-19 or had symptoms indicative of COVID-19. The documents were read to study participants who were not literate, and they indicated their consent by signing with their initials or an X. The study participants were allotted identity numbers during the selection process to keep their identity anonymised. Participants were informed of their rights to participate and that they could withdraw from the study at any time. Participants were given a gift (cost: USD 15) as a reward for their participation on the study.

### 2.4. Data Collection

The data was collected during November 2020 to April 2021. The twelve FGDs, six in each area, were conducted in a large well-ventilated community space to optimize air flow and physical distancing (i.e., participants were spaced two meters apart) to reduce the risk of COVID-19 transmission according to the Government COVID-19 restrictions at the time of data collection. Each FGD lasted up to one hour and was facilitated by two research assistants for quality assurance, discussion facilitation and audio recording. The research assistants were fluent in both the local languages (Urdu and Pashto) and were trained by a senior researcher from the Institute of Health Professions Education and Research, Khyber Medical University in focus group methodology and COVID-19 awareness/personal protective equipment use prior to commencing data collection. Topic guides (Appendix A and Appendix B) were used to structure the FGDs and aimed to explore the community’s experiences and willingness to consume biofortified flour. The topic guides were designed and discussed with the multidisciplinary research team.

### 2.5. Data Protection

FGDs were audio recorded using password protected and encrypted Dictaphones. The recordings were saved as password-protected digital audio files on a secure server at Khyber Medical University. The original recordings were then deleted from the encrypted Dictaphones within one day of the FGD. Digital audio files were transferred to UCLan using Microsoft OneDrive. UCLan was the Data Hub for the project, and data was securely stored in accordance with UCLan data protection and ethics protocols.

### 2.6. Data Analysis

Audio recordings were transcribed and translated into English by a professional agency in the UK (Language Insight, Lancashire, UK). FGD facilitators double-checked the transcripts for accuracy. All participant names or identifying information were removed to maintain participant anonymity. Qualitative data was coded and thematically analysed concurrently by researchers in Pakistan and the UK using qualitative data analysis software NVivo version 11. For data analysis, we followed the six-step to thematic analysis proposed by Braun and Clarke following an inductive, semantic, contextualist approach [22].

The first two authors (one UK based and one based in Pakistan) independently familiarised themselves with the data, generated initial codes, searched for themes and defined and named initial themes. Candidate themes and interpretations were presented to the wider team and compared. Any differences were resolved through discussion and feedback. Upon feedback from all authors, themes were refined and transcripts re-read and re-coded. The data extracts that best reflected the themes were chosen as illustrative examples in this article. The data files were shared and the third and fourth authors provided suggestions and feedback to further remodify them. The first and second authors shared and presented the final data to all authors for their agreement.

## 3. Results

### 3.1. Participant Characteristics

A total of 12 FGDs (four FGDs with male community members, four FGDs with female community members and four FGDs with male Jirga members) were conducted. Each FGD consisted of six participants. Due to conservative community dynamics, female participants of the FGDs had either familial relationship to the male FGD participants or to the male study project manager. All 72 people (24 women and 48 men) that were initially invited to participate in the FGDs accepted and none declined.

### 3.2. Study Findings

Five key themes were identified: contribution to food security, better sensory and baking properties than local flour, perceived health benefits of the RCT flour, willingness to pay for the flour and the importance of trusted promoters/suppliers.

The findings relate to consumption of study flour, whether this was ‘control’ flour or ‘biofortified’ flour. The trial was double-blinded and neither the participants nor researchers knew which type of flour they had consumed.

#### 3.2.1. Contribution to Food Security

Participants described positive perceptions relating to the free distribution and supply of flour within their community in the light of their low-income status.

“Nowadays there is a lot of poverty, and a bag is for 1200 (USD 6.81) or 1300 (USD 7.38) Rupees, but we receive this thing (flour) here.”

(A Jirga member)

The participants living in this rural community had limited financial resource to purchase a varied diet and viewed the provision of flour as a way to mitigate this.

“We are poor people, we can’t afford fruit, we eat this flour which improves our children, and their health improves as well, and it is good for the rest of the young ones and elders as well. There isn’t any fruit and so we just consume this flour, that is why it is good for us. (Another woman) We bake the bread and we eat it with joy because this is a poor country [area], no one can afford meat, so it is just the flour we consume it and we consume it eagerly.”

(A woman)

However, some female participants perceived the distribution to be insufficient to satisfy the needs of their entire family:

“They give me 3 bags which are not enough for a month for me, so I buy 2 small bags, we are 12 to 13 people (family members) 3 bags are not enough for a month for me. We have to eat other flour because this flour isn’t sufficient for me.”

(A woman)

The provision of free flour had a positive impact on household finances and enabled some participants to use the money they saved to purchase other foods or for alternate purposes:

“We are very happy for receiving the flour. We pay back the money that we save by receiving this flour to those we had borrowed from. So, this scheme saves money for us and keep our oven fine (chuckling), our stomach linings are good with it. We can’t afford fruit due to the poverty. All these people are poor, and we are very grateful for receiving this”.

(A woman)

Some participants highlighted the importance of good local storage facilities in order to ensure the continuous distribution of biofortified flour. Disruptions in flour availability meant that individuals had to rely on borrowing additional supplies from neighbours or family members.

“There was one issue that it wouldn’t cover the whole period of month uninterruptedly when not delivered on time [so they would have to use other flour during that gap]. That issue was there in the initial months but when they arranged this store here and the goods came here then there hasn’t been any shortage until this month, in this month there is a shortage, our flour is finished and we (borrow) it from one and other home.”

(A jirga member)

Any negative impact on food security due to COVID-19 pandemic-related restrictions appeared to be alleviated by participation in the study.

“The conditions deteriorated very much during the Corona, everything was affected yeah, but we were happy that at least we have been receiving this one relief, our food was good.” (A jirga member). Another Jirga member said, “There was a lot of difficulty, there was shortage for some time, the vehicles were banned absolutely, and if this flour were not available people would have had a big problem.”

(A jirga member)

There was a perception among participants that the project logistics team efficiently managed the distribution during the pandemic.

“We wouldn’t go as a big crowd, they would give us a schedule telling us this is when your turn is, so there would be no crowd created. Because of that the process was a bit slow but other than that we would receive a call on time to let us know that your share has arrived. So, it was handled well during the pandemic (A jirga member). No we have not, we received it regularly on time.”

(Another jirga member)

#### 3.2.2. Better Sensory and Baking Properties Than Local Flour

The participants were satisfied with the flour they received, commenting that it was cleaner, had better fermentation properties and produced better bread than the flour that they bought in their local market.

“When the bread of the bazaar [flour] cools down for a while then it turns into something like a rubber, it becomes firmer. But this flour becomes like (our very own) wheat. Its colour is also good, white colour. This flour is much better flour than the bazaar one; our females tell us this is very easy for them in fermenting, they say wetting it is very easy for them (A man). sir, there is absolutely difference between this one and the bazaar one.”

(Another man)

Some of them expressed that there was consistency in the quality of the flour received, whereas the flour available in the market would sometimes be mixed with other things or that they did not believe that they received what they had paid for.

“There is a big difference; the big difference is that there is no idea what is getting mixed into the other flour. The cold (old) bread is also mixed into it and this is something I saw with my own eyes in a flour mill.”

(A man)

#### 3.2.3. Perceived Health Benefits of the Flour

As participants of the RCT all FGD participants consumed either control or biofortified flour. Despite being blinded to which arm of the trial they had been allocated to, most participants perceived the study flour to have health benefits, stating that since they started consuming the flour provided their families had fewer stomach problems, fewer respiratory infections and more energy

“Our throats used to have a problem all the time, all the time but since we have started this flour, we got rid of that (throat problem). When we were eating the common flour, we had to take stomach tablets in the morning but since we have started this flour, we don’t have any stomach problem, our stomachs are fine. Since we have started, we didn’t have any throat issue and even we didn’t have a flu. All of us and our children became stronger. There isn’t any (problem) with it, and we want this project to run further because it is beneficial, and it has benefited us very much.”

(A man)

Both male and female participants expressed concerns that their health may deteriorate when the programme came to an end and they stop receiving the flour.

“We hadn’t eaten flour like this before. Stomach and abdomen and so on also haven’t got any problem with it. But the issue is that they say that after one and a half month or two months, this is going to finish so it means we will go back to the situation we had been in before. So, our request is that whoever is behind this decision they would listen to us and so they keep this continuing for some more time.”

(A man)

“We are in good health with this, but we don’t know what to do as now our health is going to deteriorate.”

(A woman)

#### 3.2.4. Willingness to Pay for the Flour

The perceived health benefit was one of the reasons participants gave for their willingness to buy biofortified flour once the project had ended.

“I will certainly buy it because we have used it and we know it. It helps our health and brings energy therefore we will definitely be buying this flour. (A man) It is absolutely like that, I mean, if we stopped receiving the flour, and it were available at the market and if it is in a shop in the market then we will be buying it as we know the benefits of it.”

(Another man)

An important factor guiding the community member’s decision to buy a particular type of flour related to its price. The participants were willing to pay a slightly higher price for biofortified flour due to its quality and perceived health benefits.

“We are poor people… so the price should be reduced for us, but when you bear in mind it’s better quality then if it is 200 (USD 1.14) or 100 [rupees] (USD 0.57) more expensive, it will be fine. It is good.”

(A woman)

“The reason we will be buying this one is… even if this is ten or eight rupees (USD 0.057) more expensive then so be it as all praise be to Allah we are protected from illness and problems that children would have... We are labourers and we would miss our labour work [for taking our children to a doctor]... So we do not have to bother about those problems anymore and that’s why we are using this flour, so if this flour comes out in the market by the grace of the holy Allah, then we will be using it.”

(A man)

Some of the participants said that they will continue buying biofortified flour as a consequence of family demand.

“In terms of baking, we ask our women about (and they say) it doesn’t get over yeasted, and they say this (flour) is good whether it is baked in a saucepan or in an oven it comes up with the same beautiful bread. So, our family will tell us to bring this one and we will certainly bring it. When we do our labour work then we will certainly buy this flour bag.”

(A jirga member)

The participants hoped for the flour to continue to be available after the programme ended, and some expressed their willingness to grow the biofortified variety on their land.

“We request the government of Pakistan through [NGO working in the community] to make this flour available to the whole of Pakistan, particularly to [Area] and more particularly to the [District] (multiple voices) yes.”

(Men)

“We are ready to grow it in the fields we have and our own land, or the lands that we have leased.”

(A jirga member)

#### 3.2.5. The Importance of Trusted Promoters/Suppliers

An important aspect relevant to future biofortification scale up activities related to the importance of trust between community members and the providers of the flour. This trust was also important in the context of the intervention trial where participants were asked to provide blood samples to monitor health status. Participants reported that some community members who had not participated in the trial were sceptical about the programme and had criticised their neighbours for agreeing to take part in the study.

“They do say and ask questions that ‘you are giving blood which affects your children later on, and they [the researchers] want indecency in the society, this is from the foreign countries’, so they say this sort of thing.”

(A man)

However, these participants believed that such comments were linked to feelings of jealously, adding that the comments did not concern them since they trusted the NGO and the individuals running the programme and perceived it to be of benefit to them.

“As far as people are concerned, those who don’t receive it and they are not in this then they do make dramas about this in the neighbourhood and vicinity [saying] this is this thing from the English people, this is that thing of the English people, but we don’t believe it because the flour benefit us.”

(A man)

“We know [member of staff] very well … I mean we know all of them so, God willing, they will not do anything wrong to exploit us and God willing they will work for our benefit as long as this programme runs.”

(A man)

“Everyone has their own opinion, yeah, and they behave according to that. But this flour didn’t give us any harm, on the contrary it benefited us. Loss and gain is in the hands of Allah but human does something with good intention. They [NGO working with the community] are working with good intention. Allah will give them a reward.”

(A man)

Some of those who had expressed some initial mistrust had changed their perception during the course of the programme, expressing their wish to be included in the study.

“They say good things and also bad things as well, however now they don’t say bad things, they say good things. (A man) Now all of them have become assured about this thing and they say if there is a chance for them to be admitted into.”

(Another man)

## 4. Discussion

This qualitative study explored the experiences and attitudes towards the study flour among households and Jirga from communities participating in the BiZiFED2 RCT [8]. Understanding the potential acceptability of Zn biofortified flour is key to the success of biofortification programmes and their potential for scale-up [14]. There was no adverse comments about the quality or performance of the study flour from FGD participants and there was overall satisfaction with the flour received during the BiZiFED2 trial. This finding suggests that adherence to the study intervention was likely to be high and aids our interpretation of the main RCT outcomes. The findings are also consistent with a previous study conducted in Pakistan in which participants tasted and rated chapati produced with biofortified wheat and conventional wheat very similarly in terms of sensory characteristics [23]. While differences in the scores were minor, Rizwan and colleagues found that the participants gave better scores to the chapati produced with biofortified wheat in terms of appearance and aroma while the conventional variety was scored slightly higher in terms of taste and texture. Participants in the current study described the flour they received as having good fermentation, taste and texture properties when compared to the flour available in the local markets. When making bread with the study flour, participants reported that it had good sensory characteristics, such as a sweet flavour, and that it remained fresher for longer in comparison to that available from the local market. This finding is important when considering the design of future intervention and evaluation studies, suggesting that both control and biofortified grains should be grown and milled similarly if participants are to remain blind to treatment allocation. The preference for study flour over market-bought flour may have been due to the short time between milling and distribution, or due to differences in milling practices, packaging, storage or re-packaging practices of the vendors and further studies should be conducted to explore this.

Participants reported that they would be happy to buy biofortified wheat if it were available in the local market. Consistent with previous studies [13,17], participants cited the perceived health benefits of the project flour, such as fewer stomach problems, as reasons for choosing to purchase it. The participants had limited opportunities to access a varied diet and viewed the provision of flour as a way to mitigate this This suggests that health promotion messages may form an important component of strategies to scale-up biofortified wheat in similar settings, despite very low levels of educational attainment and literacy. Nutrition education programmes are an important component of scaling-up vitamin A biofortified orange-fleshed sweet potato [24], orange maize [25] and yellow cassava [26], however high Zn-wheat differs in that it is an invisible property. This may reduce some barriers to acceptance while also making it harder to promote through social marketing campaigns, although the latter may still work for subsistence value chains or where consumers have strong trust in the provenance of the flour for example.

Unsurprisingly, participants also emphasised the importance of affordability and price. Participants who had access to land were eager to receive seed for the biofortified wheat variety so they could cultivate it themselves. Studies to assess biofortification costs are required, including additional production costs (e.g., for Zn-fertiliser), marketing and social behaviour change programmes and monitoring costs. In turn, affordability needs to be assessed including for the poorest households, and this can inform how to make scale-up sustainable.

Overall, there was good acceptance of the BiZiFED2 RCT within the community that participated in the trial. This acceptance may be attributed in-part to the relationship of support and trust that the Jirga and the collaborating NGO have established with over 20 years of working in this area. Jirga members, teachers, medical doctors and religious leaders are seen as trusted members of the community and during the preparation for the trial, Jirga members expressed their feeling that it was part of their role to raise awareness about the study and provide reassurance about the project to the community [17]. These findings highlight the importance of trusted relationships and understanding the dynamics of social structures within a community when conducting research and scale-up activities. Despite the existing trust between the research team and the community, negative rumours about the trial and the flour did occur including from community members who were not part of the BiZiFED2 trial. This indicates the need to sensitise and engage communities beyond trial participants. The observation that fears and negative rumours diminished over time suggests that trials and scale-up programmes may benefit from pilot phases with the explicit aim of building community trust.

A major strength of this study was its in-depth approach, generating rich, qualitative data from a relatively large number of participants including men, women and Jirga in the community. There were, however, some limitations to our study. BiZiFED2 was a blinded trial and the FGDs included individuals from control and intervention households. Therefore, participants were only able to report on the attributes of ‘study flour’ and there was no way to distinguish differences between the responses of consumers of control and biofortified flour. Furthermore, participant responses may have been influenced by the receipt of free flour which may have led them to exaggerate positive aspects of the programme and the study flour. Issues of consumer acceptability outside a research setting should be investigated further. Likewise, participants were drawn from households engaged in the BiZiFED2 RCT and Jirga members from respective communities, and the opinion of these participants may not be representative of the wider community, including those who were not selected to take part in BiZiFED2 RCT, or those who declined to participate. Nonetheless, the findings still present useful information and learnings to inform future research studies and programmes that seek to scale-up biofortified wheat in Pakistan.

In this study, we assessed attitudes towards the flour provided in a biofortification trial from a consumer perspective. Further research should explore the perspectives of producers and potential vendors, including their current practices and beliefs, attitudes, potential challenges and motivators towards producing and selling biofortified wheat.

## 5. Conclusions

Our study found that there was good acceptability amongst the community towards the flour provided in the BIZIFED2 RCT, with participants reporting perceived health benefits and good cooking/sensory qualities. Participants felt that the flour was of better quality in comparison with local wheat flour. The distribution of flour was also welcomed and perceived both as a source of food security and economic support and in this low-resource setting. Our findings may be used to inform future research and scale-up programmes for biofortified wheat in Pakistan.

## Data Availability

The datasets used and analysed during the current study are available from the corresponding author on reasonable request.

## References

[1] Wessells K.R., Ouédraogo Z.P., Rouamba N., Hess S.Y., Ouédraogo J.-B., Brown K.H. (2012). Short-Term Zinc Supplementation with Dispersible Tablets or Zinc Sulfate Solution Yields Similar Positive Effects on Plasma Zinc Concentration of Young Children in Burkina Faso: A Randomized Controlled Trial. J. Pediatr..

[2] Kumssa D., Joy E., Ander E.L., Watts M., Young S.D., Walker S., Broadley M.R. (2015). Dietary calcium and zinc deficiency risks are decreasing but remain prevalent. Sci. Rep..

[3] Gupta S., Brazier A.K.M., Lowe N.M. (2020). Zinc deficiency in low- and middle-income countries: Prevalence and approaches for mitigation. J. Hum. Nutr. Diet..

[4] Mayo-Wilson E., Junior J., Imdad A., Dean S., Chan X.H., Chan E.S., Jaswal A., A Bhutta Z. (2014). Zinc supplementation for preventing mortality, morbidity, and growth failure in children aged 6 months to 12 years of age. Cochrane Database Syst. Rev..

[5] King J.C., Brown K.H., Gibson R.S., Krebs N.F., Lowe N.M., Siekmann J.H., Raiten D.J. (2015). Biomarkers of Nutrition for Development (BOND)—Zinc Review. J. Nutr..

[6] UNICEF (2019). National Nutrition Survey 2018: Key findings report. Nutrition Wing Ministry of Health Services, Regulation and Coordination.

[7] Brazier A., Lowe N., Zaman M., Shahzad B., Ohly H., McArdle H., Ullah U., Broadley M., Bailey E., Young S. (2020). Micronutrient Status and Dietary Diversity of Women of Reproductive Age in Rural Pakistan. Nutrients.

[8] Lowe N.M., Zaman M., Moran V.H., Ohly H., Sinclair J., Fatima S., Broadley M.R., Joy E.J.M., Mahboob U., Lark R.M. (2020). Biofortification of wheat with zinc for eliminating deficiency in Pakistan: Study protocol for a cluster-randomised, double-blind, controlled effectiveness study (BIZIFED2). BMJ Open.

[9] United Nations Goal 2 Zero Hunger. https://www.un.org/sustainabledevelopment/hunger/.

[10] Lowe N.M. (2021). The global challenge of hidden hunger: Perspectives from the field. Proc. Nutr. Soc..

[11] Marques E., Darby H., Kraft J. (2021). Benefits and Limitations of Non-Transgenic Micronutrient Biofortification Approaches. Agronomy.

[12] Osendarp S.J.M., Martinez H., Garrett G.S., Neufeld L.M., De-Regil L.M., Vossenaar M., Darnton-Hill I. (2018). Large-Scale Food Fortification and Biofortification in Low- and Middle-Income Countries: A Review of Programs, Trends, Challenges, and Evidence Gaps. Food Nutr. Bull..

[13] Talsma E.F., Melse-Boonstra A., Brouwer I. (2017). Acceptance and adoption of biofortified crops in low- and middle-income countries: A systematic review. Nutr. Rev..

[14] Saltzman A., Birol E., Bouis H.E., Boy E., de Moura F.F., Islam Y., Pfeiffer W.H. (2013). Biofortification: Progress toward a more nourishing future. Glob. Food Secur..

[15] Lowe N.M., Khan M.J., Broadley M., Zia M.H., McArdle H.J., Joy E.J.M., Ohly H., Shahzad B., Ullah U., Kabana G. (2018). Examining the effectiveness of consuming flour made from agronomically biofortified wheat (Zincol-2016/NR-421) for improving Zn status in women in a low-resource setting in Pakistan: Study protocol for a randomised, double-blind, controlled cross-over trial (BiZiFED). BMJ Open.

[16] Ohly H., Broadley M., Joy E.J.M., Khan M.J., McArdle H., Zaman M., Zia M., Lowe N. (2019). The BiZiFED project: Biofortified zinc flour to eliminate deficiency in Pakistan. Nutr. Bull..

[17] Mahboob U., Ohly H., Joy E.J.M., Moran V., Zaman M., Lowe N.M. (2020). Exploring community perceptions in preparation for a randomised controlled trial of biofortified flour in Pakistan. Pilot Feasibility Stud..

[18] Zaman M., Afridi G., Ohly H., McArdle H.J., Lowe N.M. (2020). Equitable partnerships in global health research. Nat. Food.

[19] Ohly H., Lowe N., Broadley M., Joy E., Khan M.J., Mcardle H., Medhi R., Shahzad B., Zaman M. (2019). Exploring socio-cultural aspects of the foods environment: Study perspectives from Pakistan. UNSCN Nutr..

[20] TRUST Consortium (2018). Global Code of Conduct for Research in Resource-Poor Settings. https://www.globalcodeofconduct.org/wp-content/uploads/2018.

[21] Lowe N.M., Zaman M., Khan M.J., Brazier A.K.M., Shahzad B., Ullah U., Khobana G., Ohly H., Broadley M.R., Zia M.H. (2022). Biofortified Wheat Increases Dietary Zinc Intake: A Randomised Controlled Efficacy Study of Zincol-2016 in Rural Pakistan. Front. Nutr..

[22] Braun V., Clarke V. (2006). Using thematic analysis in psychology. Qual. Res. Psychol..

[23] Rizwan M., Zhu Y., Qing P., Zhang D., Ahmed U.I., Xu H., Iqbal M.A., Saboor A., Malik A.M., Nazir A. (2021). Factors Determining Consumer Acceptance of Biofortified Food: Case of Zinc-Fortified Wheat in Pakistan’s Punjab Province. Front. Nutr..

[24] Laurie S., Faber M., Adebola P., Belete A. (2015). Biofortification of sweet potato for food and nutrition security in South Africa. Food Res. Int..

[25] Simpungwe E. (2017). Orange maize in Zambia: Crop development and delivery experience. Afr. J. Food Agric. Nutr. Dev..

[26] Bechoff A., Chijioke U., Westby A., Tomlins K.I. (2018). ‘Yellow is good for you’: Consumer perception and acceptability of fortified and biofortified cassava products. PLoS ONE.

